# Educating the Public Health Workforce: A Scoping Review

**DOI:** 10.3389/fpubh.2018.00027

**Published:** 2018-02-19

**Authors:** Donghua Tao, Connie J. Evashwick, Michal Grivna, Roger Harrison

**Affiliations:** ^1^Saint Louis University Medical Center Library, St. Louis, MO, United States; ^2^Milken Institute of Public Health, George Washington University, Washington, DC, United States; ^3^College of Medicine and Health Sciences, United Arab Emirates University, Al-Ain, United Arab Emirates; ^4^School of Health Sciences, University of Manchester, Manchester, United Kingdom

**Keywords:** public health workforce, public health workforce training, public health workforce pedagogy, literature search, public health workforce education, health workforce worldwide, global health workforce

## Abstract

The aim of this scoping review was to identify and characterize the recent literature pertaining to the education of the public health workforce worldwide. The importance of preparing a public health workforce with sufficient capacity and appropriate capabilities has been recognized by major organizations around the world (1). Champions for public health note that a suitably educated workforce is essential to the delivery of public health services, including emergency response to biological, manmade, and natural disasters, within countries and across the globe. No single repository offers a comprehensive compilation of who is teaching public health, to whom, and for what end. Moreover, no international consensus prevails on what higher education should entail or what pedagogy is optimal for providing the necessary education. Although health agencies, public or private, might project workforce needs, the higher level of education remains the sole responsibility of higher education institutions. The long-term goal of this study is to describe approaches to the education of the public health workforce around the world by identifying the peer-reviewed literature, published primarily by academicians involved in educating those who will perform public health functions. This paper reports on the first phase of the study: identifying and categorizing papers published in peer-reviewed literature between 2000 and 2015.

## Introduction

The World Health Organization (WHO) has a defined list of activities necessary to effectively keep the public healthy, continue to improve population health, and reduce global inequalities (2). As the enactors of these functions, the public health workforce is widely recognized as critical to promoting the health of communities within and across nations. The WHO (1), Institute of Medicine (3), the Centers for Disease Control and Prevention of the USA (4), and the Harvard School of Public Health and China Medical Board (5) have all highlighted the importance of educating the public health workforce to continue to improve health around the world. Recent recognition of the importance of the social determinants of health (6) has called even more attention to the need for comprehensive training of all those with primary or secondary public health roles.

Preparing those who will promote the health of the public is challenged by the unresolved, if academic, controversy about whether public health is a distinct discipline or not (7). “Health professionals who perform public health functions” and “public health professionals” are considered distinct by some and singular by others. A recent report by two universities in the United States (US) (8) explains how different sources define the public health workforce differently and the resulting challenge this poses in getting an accurate count of practitioners and the related issue of calculating and ultimately creating optimum capacity.

Regardless of the methods used to define and enumerate its size, the “public health workforce” is huge and diverse (9). As such, educators have attempted to address effective methods to support workforce needs in terms of education and skills development. A wide array of competency frameworks exists which aim to link education to effective practice, both discipline-specific (10, 11) and interdisciplinary public health (12). Diversity also prevails across and within countries (13), with different organizations responsible for public health education and training. Some education and competency frameworks are linked to professional standards (14); others to accrediting agencies (15). Some organizations have recently come together to promote coordination of competencies across disciplines (16) and in support of a global perspective for international education and collective action on the ground (17). Arguably, this level of diversity in terms of workforce right through to local and global quality assurance sharply contrasts with other key health-related occupations, such as medicine, nursing, and dentistry. Thus, a fragmented and inconsistent approach to public health manpower planning and education seems endemic throughout the global public health community.

Moreover, for several decades, there has been a strengthening in using evidence-based principles to ensure effective design and delivery of education for medical, dental, and nursing students despite the methodological challenges it presents (18–21). Yet until recently, no specific journal was dedicated to the pedagogy for educating the public health students and future workforce. The complexity of public health as a discipline, along with a wide variation in training provision, makes it difficult to determine the strengths and weaknesses of different teaching practices for public heath students in higher education.

The fragmentation about how and in what to train the public health workforce becomes particularly detrimental in confronting an increasingly global world. Infectious diseases once contained in local regions now spread rapidly across continents. Advocacy campaigns that previously might have been local, regional, or at most, national, now quickly “go viral” through the use of social media. From the business perspective, multi-national trade deals are common; yet multi-national collaboration to prepare the public health workforce falls short. Accreditation, licensing, visas, foreign clinical privileges, and other barriers prevent health professionals, including those serving public health functions, in one country from working in another country, at least not without meeting extensive legal and training requirements.

This diminishes opportunities to create a global public health workforce with the capacity to address cross-national emergencies and the competencies required for global tasks. French et al. recommend “a systems approach” to education and manpower planning (5). It is unclear how other countries, governments, universities, and public health professional bodies are ensuring that workforce capacity building fits current needs and future projections.

*The purpose of this study was to begin to build a basis for discussion about cross-national public health workforce education by identifying who is teaching what, where, and with what competency or learning objective framework*. Phase 1 of the study is the scoping review reported here. From this base, subsequent analyses will examine the content of the literature and the implications for current and future worldwide education of the public health workforce.

## Materials and Methods

Scoping review has become an increasingly common tool to provide a descriptive overview of the reviewed materials as the body of literature worldwide has grown and become more accessible (22). A scoping review can be of particular use when the topic has not yet been extensively reviewed or is of a complex or heterogeneous nature (22), which is appropriate for public health workforce issues. This scoping review found and characterized the peer-reviewed literature from around the globe between 2000 and 2015 pertaining to the education of those providing public health functions at a professional level.

### Search Process

**Figure 1:**
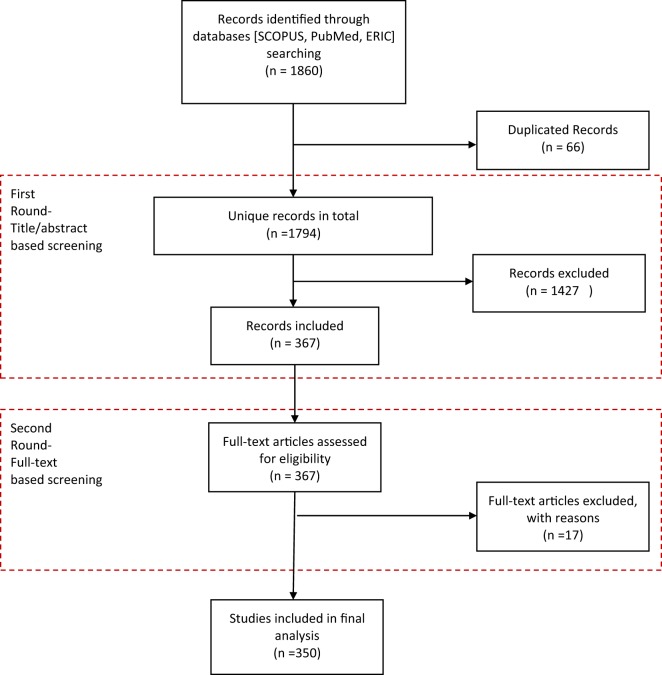
Flow chart of literature review process.

Figure 1 shows the search process. Eligible studies were first identified from a search of the major databases of health and education-related publications: Scopus, PubMed, and ERIC. The search terms were developed from essential functions of public health and broadened with the use of additional medical subject headings, combined with Boolean operators. United Kingdom (UK) and US variations in spelling were accommodated. Main free-text search terms included Public Health, Environmental Health, Occupational Health, Biostatistics, Health Behavior, Health Education, Health Services Administration, Health Policy, Population Health, Health Regulations, Pedagogy, Competency, Learning outcomes, Accreditation, Certificate and licensure, Competencies, Interprofessional, and Service Learning. Only English language publications were included from January 1, 2000, to December 31, 2015.

The time frame 2000–2015 was selected as relevant because it is during this recent era that the use of competencies has come into being as a way to shape and evaluate education (23) and that worldwide communication methods, particularly the Internet, has fostered globalization, including the delivery of education (24). When the study was initiated in 2016, the last year for which complete data were available was 2015.

### Inclusion Criteria

Given the diverse nature of public health roles, the review included journal articles that matched the WHO definition of essential activities for the public’s health (2) and/or for which a published framework of educational competency or additional defined essential content existed either as a main or subspecialty. Primary disciplines, such as medical, nursing, and dental undergraduate degrees, were included along with public health, as were sub-specialties of public health, such as epidemiology, health administration, and health education. Definitions and competency frameworks were current through the end of 2015.

Given that the focus was on “professional” public health providers, level of education was considered as that which provides a terminal degree leading to employment in public health in the relevant country. For example, in the US, a master’s of public health (MPH) is the most common degree, as post-high school baccalaureate education in public health tends to take an “educated citizen” generalist outcome approach. In countries where post-high school education is 6 years and includes focused health professions education, such as China, articles deemed acceptable for the scoping review would include those covering baccalaureate curricula.

### Selection Process

The first 100 citations were used as a pilot to go over the process iteratively until the acceptable level of interrater reliability with Cohen kappa >0.8 was reached (25). The first stage of the process was based on the title/abstract review. Four reviewers formed two pairs. Two reviewers of each pair reviewed the same 100 citations independently, then met to discuss and resolve discrepancies in whether a study met the inclusion criteria or not. For the studies without reaching agreement, full text articles were reviewed to finalize the consensus on the inclusion or exclusion. Once the interrater reliability level was acceptable, the rest of the unique citations after removing 100 sample citations were divided evenly for each of four reviewers to code “Yes” with 1, “No” with 2, and “Maybe” with 3. For the citations with the “Maybe” code, two pairs of review teams were formed with different members in the pilot process to ensure the coding reliability. Each pair reviewed half of the citations with the “Maybe” code until the consensus on inclusion/exclusion was reached. A final list of inclusive citations was identified after this entire iterative process.

### Categorization Process

Data extracted from each article included authors, year of publication, country/region, discipline/program of study, and article “type.” The type of articles referred to methodology and was based on the description for eligible articles used by an international, peer-reviewed, open-access journal of public health education (26). The category of curriculum/instruction/pedagogy encompassed description of a course, description of curriculum, case study (i.e., unique situation), description of field experience, or competency framework. The category of evaluation was used for articles reporting data on formal evaluation studies assessing the outcomes of courses, curricula, or specific teaching methods.

A challenge to the study was in assigning articles to just one specific category. For example, a case study might include a formal evaluation of student learning outcomes. The authors agreed to allow two classifications of article type for each article. This explains why the number of article types is greater than the number of individual articles in the reported results. A more detailed analysis using free-text mining considered the methodologies employed, independent of article type.

## Results

As shown in Figure 1, from 1,860 citations originally identified from the searches, a total of 350 articles met the final inclusion criteria for the review. The list is available in Data Sheet S1 in Supplemental Material.

### Year of Publication

**Figure 2:**
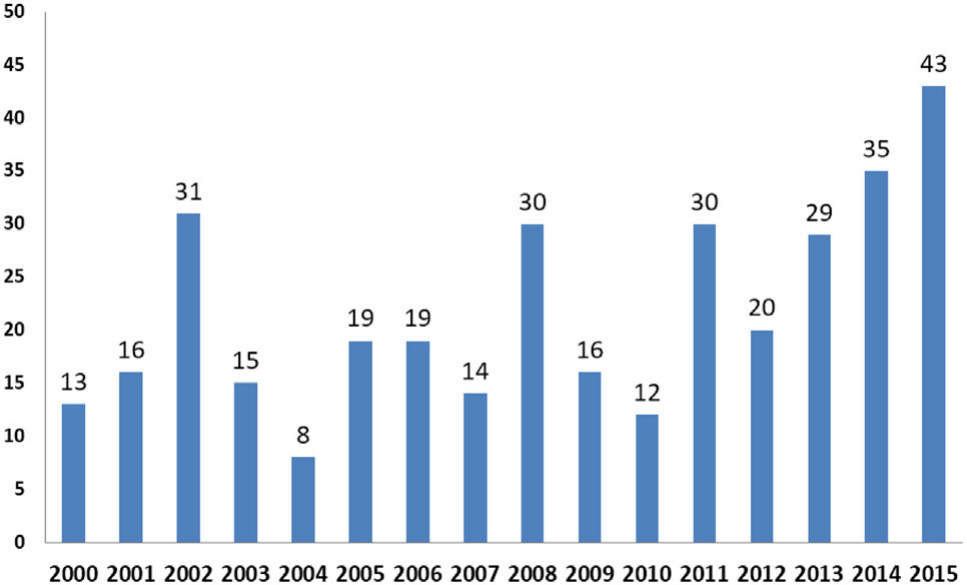
Number of articles by year, 2000–2015.

Figure 2 shows the number of articles published in each year. The distribution indicates that articles pertaining to the education of the public health workforce have steadily increased over the 15 years between 2000 and 2015.

### Geographic Distribution

Table 1 shows the geographic location of the articles. A total of 46 countries and/or regions were included in the review, and all continents were represented. The predominant countries were those in North America and Europe. Of the 350 articles, 177 focused on education pertinent to the US. Twenty-four articles focused on the UK; 17 on Canada; and 14 on Australia. Several articles described multi-national efforts, including across regions, or a global emphasis without being geographically specific.

**Table 1:** Distribution of articles by geography.

Region and country	Number	Totals
**North America**		
Canada	17	
Mexico	2	
United States	148	
Total		177
**Europe**		
Europe (broad focus)	9	
United Kingdom	24	
Italy	3	
Ireland	2	
Germany	2	
Norway	1	
Netherlands	1	
Lithuania	2	
Switzerland	3	
Poland	1	
Turkey	3	
Spain	1	
Total		52
**Eastern Europe**	2	
Croatia	2	
Albania	1	
Bulgaria	1	
Russia	1	
Total		7
**Mid-East**		
Israel	5	
Jerusalem	1	
Palestine	1	
Saudi Arabia	2	
Kuwait	1	
Total		10
**Asia**	1	
China	2	
Hong Kong	1	
India	6	
Pakistan	2	
Sri Lanka	1	
Thailand	1	
Malaysia	1	
Total		15
**Down Under**		
Australia	14	
New Zealand	1	
New Caledonia	1	
Total		16
**South America**	2	
Brazil	3	
Chile	3	
Colombia	1	
Total		9
**Africa**	3	
South Africa	2	
Kenya	1	
Total		6
**Multi-national/global**		4
Total		296
No geographic area specified		72
Two countries or regions included		3

### Discipline

As shown in Table 2, the majority of articles focused on public health as a main discipline in general (166 in total) or sub-specialties of public health (60 in total), such as epidemiology, health management, or health education. Nonetheless, articles also emanated from the disciplines of medicine, nursing, dentistry, veterinary medicine, pharmacy, social work, and others.

**Table 2:** Number of articles by discipline.

Discipline	Number of articles included in review
Public Health—General	168
Medicine	62
Public Health—Sub-Specialties	60
Biostatistics	7
Emergency Preparedness	2
Environmental Health	4
Epidemiology	15
Informatics	4
Health Education	13
Health Management/Health Admin	12
Nutrition	3
Multi-Disciplinary/Interdisciplinary	26
Nursing	13
Dentistry and Dental Hygiene	8
Veterinary Medicine	4
Social Work	4
Other	3
Pharmacy	2

### Article Type

Table 3 gives the article types of the publications. The largest proportion of article types from the original list of 350 was for curriculum, instruction, pedagogy (36%, 102), followed by evaluation (24%, 84) and perspective/opinion (15%, 53). One-in-ten or less were identified as studies primarily focused on original research (10%, 36), case study (10%, 34), “other” (6%, 23), review (4%, 16), competency (4%, 13). Less than five articles met the format of a hypothesis/theory focused article, accreditation/certification, or commentary. When regrouped into a single category of original research, evaluation, teaching method, curriculum/instruction/pedagogy, and competency, 290 articles had focused on this as a part of the primary or secondary investigation.

**Table 3:** Number of article types as the primary or secondary focus.

Article type (based on Frontiers’ categories)	Primary	Secondary	Total	%
Curriculum, Instruction, Pedagogy	102	25	127	36.3
Evaluation	55	29	84	24.0
Perspective or Opinion	48	5	53	15.1
Teaching Method	29	14	43	12.3
Original Research	36	0	36	10.3
Case Study	31	3	34	9.7
Other	11	12	23	6.6
Review	15	1	16	4.6
Competency	13	0	13	3.7
Commentary (response to previously published article)	5	0	5	1.4
Hypothesis and Theory	4	1	5	1.4
Accreditation, Certification, Licensure	1	2	3	0.9
Book Review	0	0	0	0.00

Total	350		442	

N.B. percentages add up to more than as some articles were included in more than one “article type.”

As shown in Table 4, 97 (28%, 97) articles mentioned a research design keyword in the title or abstract. The most frequent study design mentioned was cross-sectional/survey design, stated in 76% of articles (74/97), 14% (14/97) conducted interviews, and 8% (8/97) employed focus groups. One study carried out a randomized controlled trial. Some studies were double counted with more than one keyword identified (such as mixed-methods studies).

**Table 4:** Core methodologies based on text mining for key methodological terms.

Methodology	*n*	%
Randomization	1	0.29
Cross sectional	74	21.14
Cohort/longitudinal	0	0.00
Focus groups	8	2.29
Interviews	14	4.00

Total keyword(s) identified	97	27.71

*N.B. total is less than 350 as articles with non-rigorous designs were excluded, such as articles describing courses or curricula*.

Of the entire 350 articles, only 3 had a primary focus of accreditation/licensure/certification, and for 2 of these, this was the secondary topic of emphasis. The single randomized controlled trial, conducted in Canada and published in 2015, examined the benefits of learning compared with use of printed articles/resources.

### Authors

The distribution of authors was examined as an indication of the breadth of interest in the education of the public health workforce. The 350 articles were written by a total of 2,432 authors. The number of authors for an article averaged nearly 7, with a range from 1 to 17. Single author papers numbered only 16. The papers with a larger number of authors typically represented the work of multi-institutional task forces to develop competency models, such as global competencies or MPH competencies. As noted in the number of countries represented by the 350 articles, authors came from all over the world. Very few authors (less than 12) appeared on more than one paper. The most articles authored or coauthored by a single author numbered five.

## Discussion

The comprehensive review of literature investigating undergraduate and postgraduate education for the public health workforce identified 350 articles over 16 years from 2000 to 2015. The number of relevant publications increased steadily overtime, suggesting increasing recognition in what historically has been an overlooked field. Many countries and all continents had conducted studies in this area. Few can deny that public health has become a global discipline. As such, educators around the world need to ensure effective pedagogy that meets the teaching needs of their own students and incorporates application and knowledge transfer to other populations.

The current review also identified a diverse public health workforce in terms of other professions and included medicine, nursing, dentistry, veterinary medicine, social work, pharmacy, physical therapy, occupational therapy, among others. The collection of articles revealed attention being given to components of education across the spectrum of public health workers, be it as sub-activity within their wider professional grouping or as a primary public health worker.

The distribution of authorship supports the assertion that interest in educating the public health workforce is starting to become widespread. Most articles were written by multiple authors, perhaps indicating the complexity of the subject matter and the value of multiple perspectives. Many were written by coauthors representing several institutions. In contrast to biomedical research or health systems research, where centers, institutes, and program projects have a research team that produces many articles, perhaps with variation in order to first author, neither single author nor institution dominated, with fewer than a dozen authors appearing more than once. This might indicate involvement by many people, but few, if any, centers dedicated to examining the pedagogy pertinent to the public health workforce.

By far, this is the largest and most current scoping review on the topic of public health workforce education. In the seminal work by Frenk et al. (5), only 221 of 11,054 articles (2%) reviewed for education related to the “health” workforce were focused on public health; the majority were for physicians 8,069 (73%) and 2,764 (25%) nurses. This leads to the conclusion that the education of those providing public health functions receives less attention, either from its own discipline or other health professions disciplines.

As noted by Evashwick et al. (7), articles about the education of the public health workforce are difficult to find for several reasons. These include that, unlike most health professions, until recently, public health had no journal of its own devoted to pedagogy. Yet they exist for medical education (for example, the *Indian Journal of Medical Education*), nursing education (for example, the *Journal of Nursing Education*), and dental education (for example, the *European Journal of Dental Education*), to name just a few. Furthermore, alongside the more context- and community-orientated focus of public health, there is a vast array of “gray literature,” including that from government agencies, local health administrators, universities, and the third sector/non-government organizations. Consequently, the true size and scope of activity associated with educating the public health workforce is likely to be considerably bigger than that reported here and in other studies.

The results show a lack of rigorously controlled studies, either as randomized or non-randomized intervention studies. The results identified just one study of this article type. Yet this type of methodology is as well suited to examining the effectiveness of education interventions for different groups of students, as it is for a specific procedure, therapy, or policy. Moreover, it is used to inform education of other health-related disciplines (27). Despite the trend to base pedagogy on competencies, the education community as a whole seems to continue to use student grades and satisfaction questionnaires as the main markers of “effectiveness.” Other methods/theoretical evaluation designs, for example, as a comparator against benchmarks or employer demands, are not the norm. The results from the current review support cross-sectional studies being the typical evaluation method of educational interventions for public health workers through higher education. Yet the many uncontrollable biases of cross-sectional studies in clinical epidemiology remain when transferred to other forms of enquiry. To promote the evolution of educational evaluation and adoption of evolving pedagogical frameworks, funders of educational provision and accrediting authorities need to consider more tightly controlled studies to demonstrate effectiveness of teaching interventions.

The link between educational programs and workforce needs was absent from the majority of articles. Although a few articles mentioned the need for “more” public health professionals, more experts in certain sub-disciplines, or specific content (such as training in gerontology), most articles did not relate to external projections of workforce need or demand. This calls to question the basis universities use to develop training programs. If there is no relationship to the job market, how do educational programs determine the content, or indeed, which degrees to offer? Leading experts have called for a systems approach to education and manpower planning (5) and countries, such as New Zealand, are trail-blazing efforts to project new demands and revise training accordingly (28).

The absence of any literature, except for three articles, specifically mentioning accreditation, licensure, or certification raises concern. The link between education and workplace outcomes comes into question and challenges university programs educating students to join the public health workforce to relate to current and future employment needs. A couple of studies reported on feedback from students, and one study surveyed alumni for their assessment of how well prepared they were for their subsequent job demands. Although accrediting authorities typically require feedback from alumni and/or employers of their graduates on the competencies learned through a training program, these apparently are not conducted in a way rigorous enough to warrant publication, or alternatively, those conducting the studies are not motivated to turn their information into publications for external audiences. At the same time, not all countries or accrediting bodies have such requirements, but that very few articles were identified in this global review is disconcerting.

A limitation of the methods of this scoping review was that it included different programs of study rather than examining those for final degrees in public health (e.g., Bachelors and Masters in Public Health). This was an intentional decision. Focusing on a degree alone would have excluded possibly informative research on the medical, nursing, dental students, among others, included in the review, many of whom are being taught key components included in any public health degree. Moreover, degree titles, levels, and content vary across countries. An alternative approach would have been to have started the search by focusing on a particular profession/occupation group (e.g., nursing) and then searched for publications on education and public health as lower-level key words. The total number of studies included in the review might have been different using this approach. However, this path ran the risk of excluding other occupations not often regarded as participating in public health work-related activity. Yet another facet of the search criteria was using competencies. Traditional content areas, such as Maternal and Child Health, do not appear. Rather, the content would be spread across competencies, such as epidemiology or health promotion. The approach selected was chosen as the best to maximize finding relevant articles.

The scoping review did not attempt to critically assess any of the research studies, other than classifying by main study designs. By definition, a scoping review identifies and describes the breadth of literature but does not analyze the content (22). The content of the articles will be examined in detail for each category of article type as a next phase of the study. The category of curriculum, instruction, and pedagogy is of particular interest, as these are the articles that contain discussions of competencies, pedagogical techniques, evaluation outcomes, and cross-national comparisons.

As in most reviews of the literature, the search terms, their operation, and the different literature databases used all have strengths and weaknesses. Similarly, the review did not search the gray literature, including internal reports and program evaluations. No doubt, some work will have been missed, but it is unlikely this would have substantially changed the overall conclusions. Only literature published in English was used. Future review teams should include literature published in other languages, especially Spanish and those from emerging economies such as China, particularly in countries having extensive undergraduate and postgraduate education programs and emerging programs for Bachelor and Masters in Public Health degrees.

## Conclusion

Like other aspects of health-related activity, educating students so that they can effectively execute public health roles requires pedagogical research and scholarship. The current scoping review identified a widely eclectic mix of articles, predominantly from North America and Europe that examined this activity in some way. The scoping review included many descriptive reports, cross-sectional studies, few formal evaluations, and just one randomized study on teaching methods for higher education in public health. The pool of studies on this subject is relatively small over the 16-year timeframe. Nonetheless, publishing on public health workforce education has increased over the years. Moreover, the articles represent the diverse landscape of public health and its international community. Using published literature to share basic knowledge of who is being trained, by whom, with what curricula, pedagogical elements, and evaluation methods lays the groundwork for the systems approach in educating the public health workforce. A workforce trained using contemporary approaches to education and current, global-oriented content is an essential component of creating a healthy population worldwide.

## Author Contributions

CE, DT, and RH conceived this study. DT conducted the literature search and revisions. MG, RH, DT, and CE participated equally in reviewing and categorizing the articles of the literature search. MG and DT produced the graphics. RH and CE produced the tables. CE, RH, DT, and MG contributed to the writing and editing of the text.

## Conflict of Interest Statement

The authors declare that the research was conducted in the absence of any commercial or financial relationships that could be construed as a potential conflict of interest.

## Supplementary Material

Data Sheet S1 Database of articles pertaining to health workforce education.
